# Supplementary figures and images for: Microhabitat and ectomycorrhizal effects on the establishment, growth and survival of Quercus ilex L. seedlings under drought
Source: PLoS One. 2020 Jun 5;15(6):e0229807. doi: 10.1371/journal.pone.0229807 (PMC7274372; doi:10.1371/journal.pone.0229807)

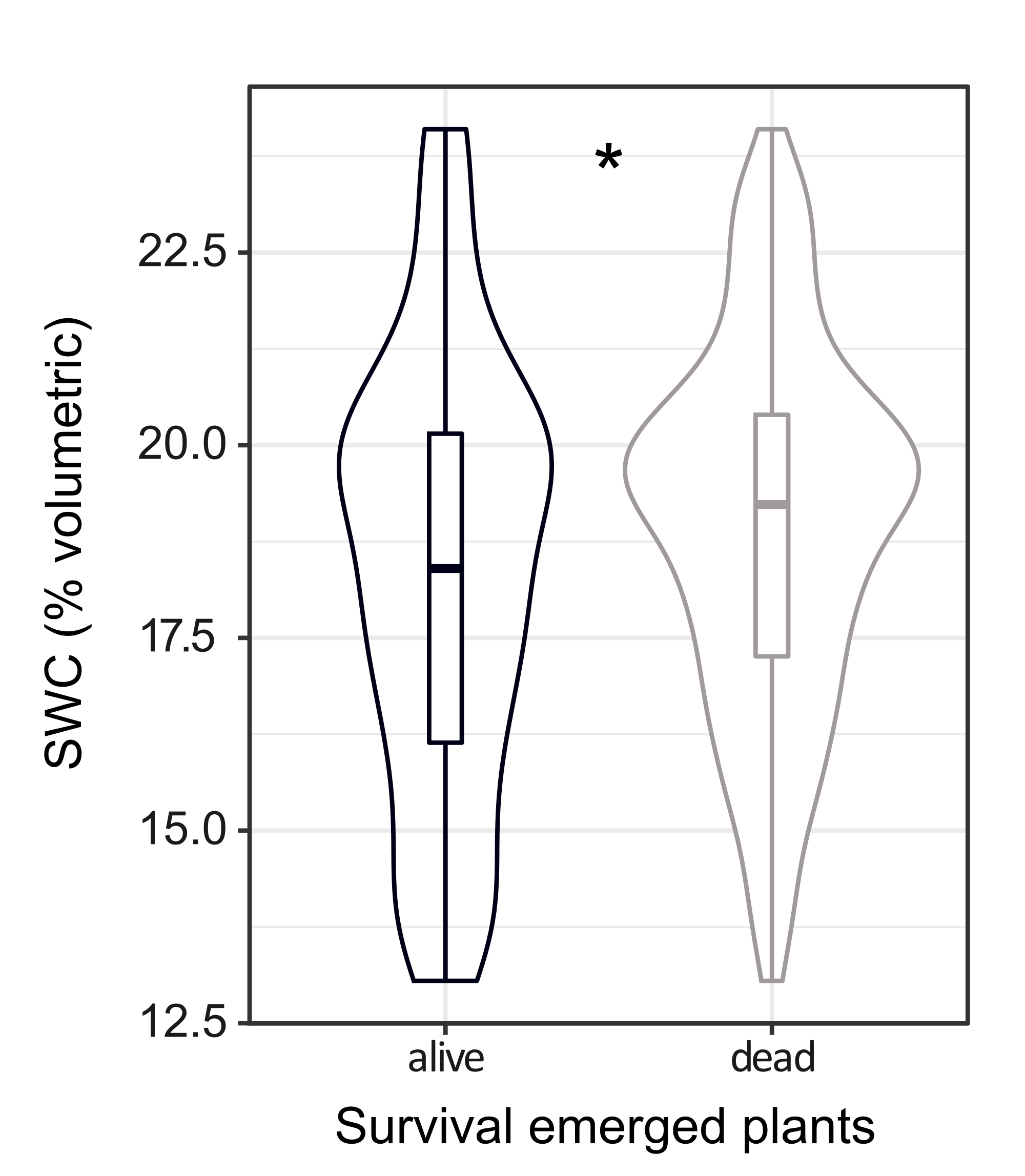

Supplement: S1 Fig — n = 391. (TIF) [file pone.0229807.s001.tif]
