# Supplementary material for: Microhabitat and ectomycorrhizal effects on the establishment, growth and survival of Quercus ilex L. seedlings under drought
Source: PLoS One. 2020 Jun 5;15(6):e0229807. doi: 10.1371/journal.pone.0229807 (PMC7274372; doi:10.1371/journal.pone.0229807)
Supplement: Attachment — Submitted filename: Response to Reviewers.docx [file pone.0229807.s003.docx]

**Reply to reviewers’ comments for the manuscript 16100657-T (“Microhabitat and ectomycorrhizal effects on the establishment, growth and survival of *Quercus ilex* L. seedlings under drought”) by García de Jalón et al. 2020**

**General response:**

First of all, we would like to thank the editor and the reviewer for the time invested in this manuscript and the constructive criticism. We took into account all the suggestions and comments, and we now present a thoroughly revised manuscript with specific focus on clarifying the methodology and the hypotheses. Furthermore, as requested by the reviewer, we present additional analyses.

------------------------------------------------------

Additional Editor Comments
------------------------------------------------------
This is a sound study of interest within this area of work. However, before publication, the authors need to clarify and justify their methodology, i.e., the use of dead seedlings, clarify their hypothesis and focus of their analysis.

A detailed point-by -point reply addressing the above-mentioned requests can be found below.

---------------------------------------------------------

Reviewer #1

---------------------------------------------------------

**Summary**
This paper examines the impact of drought, microhabitat, and ectomycorrhizae (ECM) on the germination, survival, and growth of an evergreen oak seedling. To do so the authors compared seedlings grown in a long-term precipitation reduction treatment, measuring soil water content and light availability at the microhabitat scale. The authors also examined the ECM colonization of seedlings, noting the proportion of different morphotypes. The authors found that total survival was higher under the precipitation exclusion experiment, and that seedlings in drier soils were colonized by ECM with hyphae that extended farther from the seedlings’ root tips.

**Major Issues**
**RQ1:** I was not totally sure what the authors were hypothesizing with their second hypothesis. I believe I understood the basis of the hypothesis - that microhabitat environmental factors are more important in determining seedling survival – however the phrasing of it made it unclear. The authors use the term mechanism in this hypothesis but only once more throughout the paper, I think the use of this word without introducing it earlier in the introduction is what through me as a reader off.

We totally agree with the reviewer that our phrasing was not clear. We now rephrased the second hypothesis as follows: “microhabitat conditions of water and light availability are better predictors of seedling survival than the PR treatment” (lines 27-28 and 108-109).

**RQ2:** I am curious about the use of both alive and dead seedlings for the ECM analysis. In other literature I have only seen seedlings alive at harvest used. Is it possible that using dead seedlings in this part of the analysis could alter the results, as a different suite of fungi may colonize dead seedlings? Could this be an explanation of the results that you found here? Additional information on this, including data about whether or not the ECM community differed between alive and dead seedlings would help alleviate this concern.

We understand the referee's reservations about our methodology, but we have several strong arguments explaining why we included both alive and dead plants in the ECM analyses. These arguments are exposed in detail below and are now included in the M&M in the “ECM quantification” section of the new version of the paper (lines 201-206).

Our reasons for including dead individuals in our ECM analyses are the following:

(1) at the time of harvest, the ECM colonization rate of the dead seedlings was accessible under the dissecting microscope, as it was for the alive ones. For the dead seedlings, this measurement corresponds to the mycorrhizal colonization rate at the moment of their death, and then constitutes a valuable information for the analysis.

(2) in Mediterranean ecosystems, most Q. ilex seedlings die during the first year, particularly under adult trees (see Richard et al. 2009, but see also Gomez-Aparicio et al. 2004). This dead material is an interesting source of data, and therefore it shoudn't be discarded from the analyses in our opinion. In our study, discarding dead seedlings in the ECM analysis would exclude 69 seedlings out of 128 and result in an important loss of information and statistical power.

However, as suggested by the reviewer, we re-analysed our dataset with the seedling "status" (i.e. alive or dead at the time of harvest) as an additional covariable in the ECM mixed model. This new statistical model is now replacing the previous analysis and the “Statistical analyses” section of the M&M have been modified accordingly (Lines 230-232). The statistical results do not differ from those of the previous model without the "status" as a covariable. This suggests that there is no substantial effect of the status of the seedling on the proportions of the different ECM exploration types and confirms the robustness of our initial conclusions. We also analysed the effect of the PR treatment on alive and dead individuals separately and confirmed our earlier results (higher proportions of contact exploration type in Control than in PR, and lower proportions of medium and short exploration type in Control than in PR treatment).

**RQ3:** In the results section when the authors discuss the effects of soil water content and light availability it is unclear whether these results are based upon means at the treatment level (i.e. precipitation reduction vs. control) or at the seedling level (i.e. microhabitat). As this is a key point that the paper is attempting to make, clarifying this is important in order to draw the conclusion the authors are drawing.

The fact that the SWC and GF variables where measured at the cage level was already mentioned at the lines 176-178 in the submitted version (now at lines 174-177). However, this is now also specifically mentioned in the section presenting the statistical models (lines 236-240). In addition, we now clarify that in the linear mixed models where we used SWC and GF as covariables, these variables represent the average of all measurement campaigns (6 measurement for SWC and 2 for GF). Furthermore, apart from the overall effect of PR treatment, in the new version of the paper we analysed the effect of PR treatment on SWC and GF per measurement campaign (sampling date) at the cage/microhabitat level and we added the significance values in Figures 1 and 2 and in text at lines (247-248 for SWC and 261-263 for GF).

**Minor Issues**
Overall, the paper is well written, however there were a few typos and grammatical errors throughout the paper that need to be addressed. Some clarification on figures is also necessary. I’ve listed all minor issues below:

• **RQ4:** Line 52:54 – confusing sentence

We changed into “In addition to the climatic effects, the success of tree recruitment under a forest cover is also highly dependent on microhabitat conditions [6-8] and biotic interactions, especially with mycorrhizal fungi [9,10]” (see lines 52-54).

• **RQ5:** Line 57:59 – citation?

We cited the study by Valiente-Banuet, A., & Ezcurra, E. (1991)

• **RQ6**: Line 59:61 – citation?

We cited the study by David et al. (2006) regarding rainfall interception, and the study of Franco, A. C., & Nobel, P. S. (1988) regarding the increase of competition for water of seedlings growing under canopy cover.

• **RQ7:** Line 63:65 – “with different soil moistures” strikes me as an odd way to put what they are trying to say; could overall use a better description of the two cited studies

We slightly rephrased this section trying to clarify our argument (see lines 62-66)

• **RQ8:** Line 91:94 – which exploration type is this?

It is the long exploration type. Thank you for noticing, it is now specified in the text at line 91.

• **RQ9**: Line 120 – typo, understorey

We tried to use used British English throughout the manuscript, so understorey is also acceptable.

• **RQ10:** Line 204 – Could it be the harvesting techniques caused the lack of long-distance exploration type?

We think that this is unlikely. First, because we anticipated that this can be an issue and we put a lot of effort into carefully extracting the root systems by digging around the plant as much as was possible despite the very stony soil in order to harvest the root systems as close to intact as possible and reduce the damage to the roots and the loss of ECMs. Second, Shanin et al. 2013 who analysed Quercus ilex root tips in the same experimental site also reported the complete absence of long exploration types in the 0-20 cm soil layer. This aspect is now included in M&M of the new version of the manuscript at lines 214-217.

Shahin, O., Martin-St Paul, N., Rambal, S., Joffre, R., & Richard, F. (2013). Ectomycorrhizal fungal diversity in Quercus ilex Mediterranean woodlands: variation among sites and over soil depth profiles in hyphal exploration types, species richness and community composition. Symbiosis, 61(1), 1-12.

• **RQ11:** Figure 1
o Are differences significant across every measurement point?

Thank you for pointing this out. There is a significant treatment effect on soil water content in each of the 6 measurement campaigns, and an overall treatment effect when considering all campaigns together. Each data point in Fig. 1 represents the average per cage/microhabitat at one given date. In this revision we indicate on Fig. 1 whether there are significant differences in SWC at each sampling date (lines 247-248).

o Where is data for June and July?

Unfortunately, we did not measure the SWC data during June and July. We measured in March, April, May, August, September and October. Our goal was to quantify differences in SWC among microhabitats (cages) and to verify that the effect of treatment on SWC was consistent through time, so we thought that six data points along the year would be enough. We do agree that it would have been better to collect SWC data each month, but it was unfeasible for us to go to the field more often.

• **RQ12:** Figure 2
o Are differences significant for both April and September as well as overall?

There is a significant treatment effect on GF in September (P=0.03), but not in April (P=0.16). In the new version of the paper we added the P-values significances per measurement campaign on Fig. 2 and in the text in lines 261-263.

o Should X axis label of “Day of Year” say month?

Correct, Fig. 2 was modified accordingly.

• **RQ13:** GF results (line 245:249 and Figure 2) – only measured in April and September, you mention the canopy is dominated by evergreen oaks, but are there other deciduous plants that could be impacting light availability between these two times?

Actually, there are almost no deciduous trees or shrubs in our experimental site. The scarce understorey is mainly composed by Buxus sempervirens, Phyllirea latifolia, Pistacia terebinthus and Juniperus oxycedrus. Pistacia terebinthus is the only deciduous species in our site but it does not represent an important number of individuals or an important leaf area, so we did not expect a large seasonal variation of GF. That is the reason why we thought that 2 measurements per year would be enough, since the evergreen canopy does not change much, and we were mostly interested in the differences among microhabitats than among dates.

• **RQ14:** Table 1
o Line 259 – typo “It is tested…”

Thank you, this has been removed.

o Effect of GF on germination, there is no symbol for significance, is this intentional?

Yes, there is no significant effect of GF on germination. In the new version of the manuscript we added the following sentence in the caption of table 1 at lines 278-280“…the estimates with a lack of symbol (star or plus) are not statistically significant, but the predictor was retained in the minimal adequate model.”

• **RQ15:** Line 270 – typo “it was significant lower…”

It is now replaced by “it was significantly lower”.

• **RQ16:** How are seedling mortality during germination and germination rate different?

The reviewer is correct, these are just two complementary values describing the same phenomenon. Mortality during germination was 25.7% in Control and 23.7% in PR, and germination rate in Figure 4A is 74.3% in Control and 77.3% in PR. Germination rate or survival during germination, is just 100% - mortality during germination, and the sum of the two makes 100%.

• **RQ17:** Line 273:277 – are these differences statistically significant?

No, these differences are not significant. This is now specified in the text beside the values to avoid any ambiguity (lines 289-293).

• **RQ18:** Figure 3
o You mention drought conditions, was the summer drier than a typical summer, or is this just the dry period typical of a Mediterranean climate?

The drought period represented in Fig. 3 was characterized based on the site precipitation and SWC measurements. We investigated several methods in order to determine the drought period for 2018 by using our SWC measurements under a threshold of <20% SWC, the precipitation of the site or a threshold of <40% REW for Relative Extractable Soil Water following Granier et al. (1999, Ecological Modelling, 116: 269-283). The three methods agree that the 2018 drought period goes from late June-early July to the beginning October, which is what we represented on Fig. 3. We updated the legend of Fig.3 to specify that this represents the 2018 drought based on SWC data (line 299-300). We now also mention that the drought experienced in 2018 can be considered a typical summer drought at our experimental site (lines 248-251).

• **RQ19:** Why did you not measure belowground biomass?

We did, it appears in Table 1 (root biomass) which corresponds to root biomass of the extracted root systems. However, we decided not to use it in the analysis of ECM colonization, because we could not extract the whole root system due to the very rocky nature of the soil (over 70% of the volumetric soil fraction). So we consider that our estimates of root biomass are not 100% reliable, and therefore, in order to avoid any potential bias on the ECM analysis we opted not to include this variable. This is now explained at line 195-196.

• **RQ20:** Figure 6
o Line 319 – typo “it is showed…”

Corrected!

o Overall, I found this figure to be very difficult to understand, does +1/-1 SD mean you are only using those values in that range?

In Fig 6, we display three-way interactions between numerical variables. There are two ways of displaying three way interactions of numerical variables. One way is with a three-dimensional graph. A second way is by using the prediction of the fitted model and present the relationship between two-variables at various levels of the third variable (called the moderator variable). We opted for the later approach using the “interactions” package available in R which is specifically designed for this purpose and presents the relationship at three levels of the moderator variable (-1SD, mean and +1SD of the moderator variable). This is now better explained in the legend of Fig. 6 at lines 341-342.

• **RQ21:** Table 2
o Line 327:328 – typo “it is tested…”

Deleted.

• **RQ22:** Line 338 – What basin are you referencing here?

The Mediterranean basin, it is now explicitly specified in the text in line 358.

• **RQ23:** Line 380 – can you see oomycete damage under the dissecting scope? If you can did you notice any damaged root tips while counting ECM colonization?

We observed many dead ECM root tips in many dead or alive seedlings. However, we didn’t link this observation to either oomycete damage or natural root turnover process at the time of measurements. The oomycetes hypothesis emerged during the data analysis and interpretation of the results, so unfortunately we did not specifically look for oomycetes damage at the time and currently have no data on oomycetes.

• **RQ24:** Line 399:42 – this doesn’t necessarily agree with what the results from this paper show, you found no impact of light on germination survival, and a positive impact on total survival, and no impact on emerged survival

We understand that this could be a bit confusing. Indeed our reasoning was that if there is no effect of light of germination and on the survival of emerged plants, but a positive effect of light on the total survival, this could mean that the positive effects of light occurs between germination and stem emergence. As this stage occurs before the summer drought, light is not yet detrimental to seedling survival by increasing the drought stress. And this is in line with Perez-Ramos et al. 2013 who found a positive effect of light on germination and emergence, but a negative effect of light on emerged seedlings. We change the text in the new version of the paper in lines 414-420 in order to clarify this issue.

**• RQ25:** Line 402:403 – could lower biomass in seedlings with more SWC also be due to the different morphotypes of ECM colonizing them?

We are not sure. We know from our results that seedlings with higher aerial biomass were found under drier soil conditions (Table1), and were less associated with contact ECM exploration types (Table 2). We also observed that in our study system contact ECM type has a tendency to be more present under wetter soil conditions, so it could be just a simple correlation between soil humidity and ECM contact, that just coincides with the fact that higher humidity is related to lower biomass without a causal link. Therefore, given these limitations, we deliberately refrain to not speculate about any biological explanation for the link between a lower presence of contact ECM and an increased aerial biomass of seedlings. We made this decision because in our study system 1) contact exploration type corresponds to a high diversity of species belonging to various lineages that could have different roles (positives, neutral or negatives) on growth of seedlings depending on the different contact ECM species and 2) the high patchiness of ECM diversity induces changes in ECM community composition on roots of co-occurring seedlings (see Courty et al. 2016) and make any generalization speculative.

This is now clarified in the new version of the manuscript in lines 465-471.

P.-E. Courty, F. Munoz, M.-A. Selosse, M. Duchemin, S. Criquet, F. Ziarelli, M. Buee, C. Plassard, A. Taudière, J. Garbaye & F. Richard 2016 Journal of Ecology 104(6) : 1585-1598.

**• RQ26:** Line 467:471 – this conclusion seems to come a little out of nowhere as you have not discussed any of this previously, why bring it up now at the very end of the paper?

Yes, we do agree that maternal effect wasn’t discussed before in the paper neither it was the objective of our investigation here. However, we thought that it could be interesting and complementary to our results to point out that acorn size (one of the more important variable explaining one-year old seedling survival in the article), is being reduced by drought stress suffered by mother trees, and this will have negative consequences in the recruitment apart from the microhabitat and ECM colonization effects. Therefore we removed the mention of maternal effects in our conclusion but still mention the potential impact the drought could have on regeneration through the acorn mass (lines 492-494).
